# Special Issue “Enteroviruses 2021”

**DOI:** 10.3390/v14020306

**Published:** 2022-02-02

**Authors:** Hung-Yao Ho

**Affiliations:** 1 Metabolomics Core Laboratory, Healthy Aging Research Center, Chang Gung University, Taoyuan City 33302, Taiwan; hoh01@mail.cgu.edu.tw; Tel.: +886-3-2118800 (ext. 3318); 2 Clinical Metabolomics Core Laboratory, Chang Gung Memorial Hospital, Taoyuan City 33302, Taiwan; 3 Department of Medical Biotechnology and Laboratory Science, College of Medicine, Chang Gung University, Taoyuan City 33302, Taiwan; 4 Research Center for Emerging Viral Infections, Chang Gung University, Taoyuan City 33302, Taiwan

Enteroviruses are a group of clinically relevant RNA viruses that causes human diseases. For instance, enterovirus (EV) A-71 can cause mild hand, foot and mouth disease and potentially severe neurological symptoms. Outbreaks of EV-A71 infection have occurred in various parts of world. Likewise, an outbreak of enterovirus D68 (EV-D68) occurred in 2014, causing respiratory symptoms and even acute flaccid myelitis. Though efforts have been put into studying the pathogenesis of these viruses, the complete picture of mechanisms of enteroviral pathogenesis is lacking. This Special Issue consists of six original studies that make significant contributions to our knowledge about enteroviruses.

To elucidate the molecular mechanism of enteroviral pathogenesis, virologists resort to new tools to identify the key features of genome that are essential to establishment of infection. Dutkiewicz et al. used SHAPE technique, chemical modification and lead ion-induced RNA cleavage to define the structural elements of the genomes of CVB2 and other enteroviruses [1]. Such motifs may be involved in the interactions with host cell proteins and probably in viral pathogenesis. Likewise, Yeh et al. used sequence analysis to study mutations within the genome of a specific EV-D68 strain that does not cause any disease in type I interferon receptor knockout mouse [2]. A number of mutations are localized to capsid-coding regions. Additionally, sequence analysis helps us define the genotypes and distribution of viruses circulating in particular geographical regions. Do Socorro Foro Ramos et al. [3] and Faleye et al. [4] studied echovirus and enterovirus in Brazil and the Southwest United States, respectively. To increase the fidelity of enterovirus genotyping, Gradel et al. applied the state-of-the-art nanopore RNA sequencing for the whole-genome sequencing of enteroviruses [5]. Pharmacological treatment of enteroviral infection remains a treatment option despite the availability of vaccines against certain enteroviral strains. In this regard, Ibba et al. synthesized novel 2-phenyl-benzimidazole derivative that effectively inhibited EV-A71 entry into host cells [6]. This compound can potentially reduce viral infection in vivo. 

Given the severity of the recent COVID-19 pandemic, the knowledge gained from these studies may benefit research of other RNA viruses, in particular, SARS-CoV2. 
